# CSF tap test in idiopathic normal pressure hydrocephalus: still a necessary prognostic test?

**DOI:** 10.1007/s00415-022-11168-x

**Published:** 2022-05-22

**Authors:** Alessandra Griffa, Giulia Bommarito, Frédéric Assal, Maria Giulia Preti, Rachel Goldstein, Stéphane Armand, François R. Herrmann, Dimitri Van De Ville, Gilles Allali

**Affiliations:** 1 Department of Radiology and Medical Informatics, University of Geneva, Campus Biotech-H4 3 232.080 (H4 building), Chemin des Mines 9, Case postale 60, CH-1211 Geneva, Switzerland; 2 Institute of Bioengineering, Center of Neuroprosthetics, Ecole Polytechnique Fédérale De Lausanne (EPFL), Geneva, Switzerland; 3 Leenaards Memory Center, Lausanne University Hospital and University of Lausanne, Lausanne, Switzerland; 4 Department of Clinical Neurosciences, Division of Neurology, Geneva University Hospitals and Faculty of Medicine, University of Geneva, Geneva, Switzerland; 5 CIBM Center for Biomedical Imaging, Geneva, Switzerland; 6 Kinesiology Laboratory, Geneva University Hospitals and Faculty of Medicine, University of Geneva, Geneva, Switzerland; 7 Department of Rehabilitation and Geriatrics, Geneva University Hospitals and Faculty of Medicine, University of Geneva, Geneva, Switzerland; 8 Department of Neurology, Division of Cognitive and Motor Aging, Albert Einstein College of Medicine, Yeshiva University, Bronx, NY USA

**Keywords:** Idiopathic normal pressure hydrocephalus, CSF tap test, Multimodal MRI, Reversible dementia, Prediction

## Abstract

**Objective:**

To assess whether gait, neuropsychological, and multimodal MRI parameters predict short-term symptom reversal after cerebrospinal fluid (CSF) tap test in idiopathic normal pressure hydrocephalus (iNPH).

**Methods:**

Thirty patients (79.3 ± 5.9 years, 12 women) with a diagnosis of probable iNPH and 46 healthy controls (74.7 ± 5.4 years, 35 women) underwent comprehensive neuropsychological, quantitative gait, and multimodal MRI assessments of brain morphology, periventricular white-matter microstructure, cortical and subcortical blood perfusion, default mode network function, and white-matter lesion load. Responders were defined as an improvement of at least 10% in walking speed or timed up and go test 24 h after tap test. Univariate and multivariable tap test outcome prediction models were evaluated with logistic regression and linear support vector machine classification.

**Results:**

Sixteen patients (53%) respondedpositively to tap test. None of the gait, neuropsychological, or neuroimaging parameters considered separately predicted outcome. A multivariable classifier achieved modest out-of-sample outcome prediction accuracy of 70% (*p* = .028); gait parameters, white-matter lesion load and periventricular microstructure were the main contributors.

**Conclusions:**

Our negative findings show that short-term symptom reversal after tap test cannot be predicted from single gait, neuropsychological, or MRI parameters, thus supporting the use of tap test as prognostic procedure. However, multivariable approaches integrating non-invasive multimodal data are informative of outcome and may be included in patient-screening procedures. Their value in predicting shunting outcome should be further explored, particularly in relation to gait and white-matter parameters.

**Supplementary Information:**

The online version contains supplementary material available at 10.1007/s00415-022-11168-x.

## Introduction

Idiopathic normal pressure hydrocephalus (iNPH)—the leading cause of reversible dementia in aging—is characterized by gait, cognitive and urinary impairments with ventriculomegaly at brain imaging [1, 2]. Difficulty of diagnosing iNPH with routine neurological and neuroradiological assessments explains why only 8% of patients receive disease-specific treatment [3]. INPH symptoms are unspecific and frequently found in other neurological disorders, such as Alzheimer’s disease (AD) or vascular dementia, which frequently occur as comorbidities [4]. Moreover, the treatment for iNPH relies on invasive shunt placement, thus requiring careful cost/benefit evaluation, especially in older populations [5]. These considerations highlight the importance of improving the diagnostic procedure to identify appropriate candidates for invasive shunt surgery from those with neurological disorders mimicking iNPH, or from iNPH patients with comorbidities that can interfere with reversibility. In this regard, a better understanding of the factors that underlie or hamper symptom reversibility is of primary importance.

Among the predictors of shunt surgery outcome, the cerebrospinal fluid tap test (CSFTT) has high positive predictive value [6] and is routinely used as prognostic test [7–9]. Nonetheless, the CSFTT is an invasive procedure with contraindications and patient discomfort. Moreover, the factors underlying symptom reversibility after CSFTT are not clear yet. Few studies have investigated clinical and neuroradiological correlates of CSFTT response, including cognitive scores [10], apathy [11], gait phenotype [12] and brain morphology [13], with non-conclusive results and without taking into account more advanced neuroimaging markers, such as white matter (WM) microstructure or brain functional connectivity. Although the pathophysiology of iNPH is not clear yet, different mechanisms have been proposed including periventricular axonal neurodegeneration and small vessel damage [14]. Moreover, alterations of large-scale brain functional organization have been observed, with particular involvement of the default mode network (DMN) [15], and partial functional normalization after CSFTT suggesting a role in determining outcome [16]. Integrating advanced neuroimaging methods probing iNPH pathophysiological mechanisms may identify reversible mechanisms that will eventually improve the diagnostic procedure and contribute to the prediction of CSFTT outcome, shading new light on the factors underlying short-term symptom reversibility.

Therefore, the aim of this study is to assess the feasibility of predicting CSFTT outcome from single and combined clinical (neuropsychological and gait features) and imaging (multimodal MRI) parameters in the same patient cohort. We derive brain features relevant to iNPH, including ventricle and sulcal morphology, periventricular WM microstructure, WM lesion load, blood perfusion in DMN and subcortical grey matter (GM), and DMN functional dynamics, which have been previously implicated in the diagnosis and pathophysiology of iNPH [14]. CSFTT outcome prediction is then performed using univariate and multivariable linear classifiers.

## Materials and methods

### Participants

Thirty-four iNPH patients and 48 healthy controls (HCs) were recruited at Geneva University Hospitals, between March 2017 and February 2021 according to a previously described protocol [8]. Briefly, inclusion criteria for patients were a diagnosis of possible or probable iNPH, ability to walk without assistance, and no contraindication for MRI. The diagnosis of iNPH was performed at a consensus case conference involving behavioral neurologists and neuropsychologists, and based on international consensus guidelines [1]. Exclusion criteria were the presence of an acute medical illness in the past 3 months, orthopedic disorders interfering with gait, and a diagnosis of secondary normal pressure hydrocephalus. 2 patients were excluded because of absence of post-CSFTT data; 2 patients and 2 HCs were excluded because of poor MRI data. Eventually, the study included a total of 30 iNPH patients (mean age 79.7 ± 6.3 years, 12 women) and 46 HCs (mean age 74.9 ± 5.5 years, 36 women). For completeness, we report that 8 (77.5 ± 4.5 years, 5 women) out of the 30 iNPH patients underwent ventriculo-peritoneal shunting 5.1 ± 3.2 months after inclusion in this study.

### Experimental protocol with CSFTT

iNPH patients underwent comprehensive neuropsychological and gait assessments before and 24 h after a CSFTT, which consisted in the removal of 40 ml of CSF with a 20-gauge spinal needle with the patient lying in lateral supine position. CSF levels of AD biomarkers including 42 amino-acid form of beta-amyloid, total and phosphorylated tau were measured using a double-sandwich enzyme-linked immunosorbent assay (INNOTEST, Fujirebio). HCs went through the same neuropsychological, gait and multimodal MRI assessments as patients.

### Gait assessment

Subjects were asked to walk at their self-selected speed on a 10-m walkway. Quantitative spatiotemporal gait features were recorded with a 12-camera optoelectronic system (Oqus7+, Qualisys, Sweden) and reflective markers placed on the feet (heel and 2nd toe) to compute average parameters including walking speed, step length and step width [8]. In addition, the participants performed the Timed Up and Go (TUG) test, a validated and largely used clinical test to assess mobility and dynamic balance [17].

### Neuropsychological assessment

A standardized neuropsychological test battery was administered. Executive functions, attention and memory—three dimensions impaired in iNPH [18]—were assessed with the categorical verbal fluency [19], the Wechsler Adult Intelligence Scale symbol digit modalities [20] and the French version of the Free and Cued Selective Reminding Test immediate free recall [21] tests, respectively. Apathy was assessed with the Starkstein apathy scale [22].

### Definition of CSFTT responders

Walking speed and TUG were considered as indicators of CSFTT outcome [23]. Patients were labeled as responders (RSP) or non-responders (nRSP) based on a percentage improvement after CSFTT of at least 10% in walking speed or TUG, following the cutoff defined in previous work [24–26]. This choice led to a reasonable balance between RSP and nRSP group sizes for statistical analyses, and to meaningful group-average absolute improvements in walking speed or TUG in RSP (see “Results”). Moreover, RSP–nRSP group-comparisons were repeated with an alternative cutoff of 15% improvement in walking speed or TUG (eTable1).

### Multimodal MR brain imaging

All subjects underwent an MRI session on the same day before the CSFTT, on a Siemens MAGNETOM Prismafit 3 T scanner equipped with a 64-channel head coil, including 3D high-resolution T1-weighted (T1w); T2-weighted (T2w); diffusion weighted imaging (DWI); arterial spin labeling (ASL); resting-state functional imaging (rs-fMRI) sequences (eTable 2).

### Image processing and rationale for regions of interest selection

Based on literature, we chose to focus on brain areas located in proximity to the ventricles and/or implicated in iNPH [14].

#### T1w

**Fig. 1:**
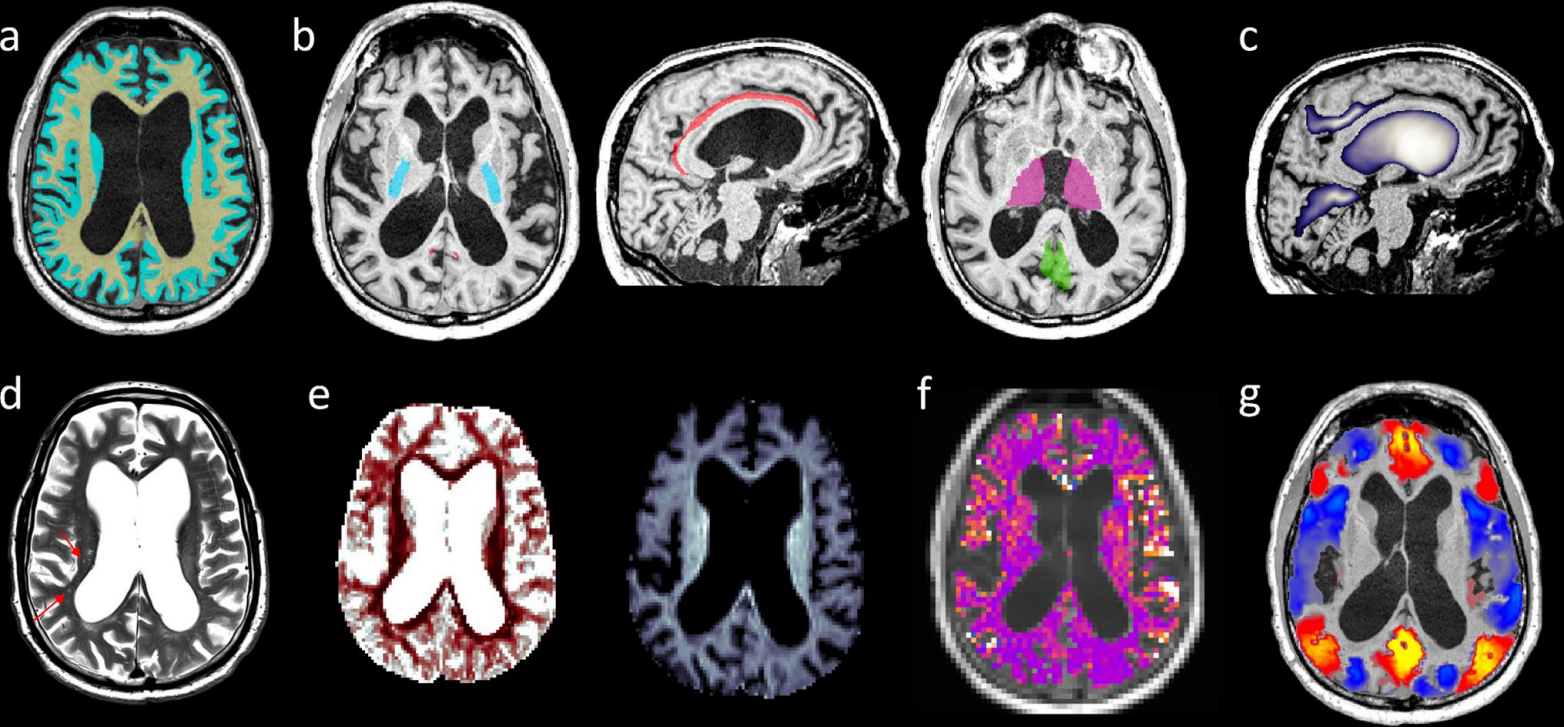
Example of multimodal MR brain imaging for a single iNPH patient. (**a**) T1-weighted images and superimposed WM (yellow) and GM (light blue) masks. (**b**) WM and GM regions of interest: posterior limb of the internal capsule (PLIC, light blue); cingulum bundle (CING, red); thalamus (THAL, violet); posterior cingulate cortex (PCC, green). (**c**) Brain morphology: the blue-to-white colormap represents probability maps for the posterior callosal marginal fissures (PCMF), the ventricles, and the calcarine fissures (CF). (**d**) T2-weighted axial slice: red arrows indicate WM hyperintensities. (**e**) ODI (dark red indicates lower ODI values, corresponding to more packed and less fanned out WM fibers) and V_ic_ (lighter blue indicates higher V_ic_ values, corresponding to larger intra-axonal volume fraction) axial slices derived from NODDI reconstruction of DWI data. (**f**) Relative blood perfusion derived from ASL data superimposed on a T1-weighted slice (yellow-white indicates higher relative perfusion). (**g**) Standardized rs-fMRI values from a single time point corresponding to PCC activation, superimposed on a T1-weighted slice [yellow–red (light blue) indicates co-activation (co-deactivation) with the PCC; only rs-fMRI values of cortical voxels are shown]

T1w volumes were segmented into GM, WM and CSF combining the outputs of state-of-the-art segmentation and spatial normalization software (FSL6.0; Freesurfer6.0.0; SPM12; ANTs2.2.0) (Fig. 1a). The volume of brain sulci (bilateral posterior callosal marginal fissure (PCMF) and calcarine fissure (CF), previously implicated in iNPH differential diagnosis [27] and prognosis [28]) and lateral ventricles was quantified based on the Brainvisa atlas v201, similarly to previous work [27] (Fig. 1c).

#### T2w

WM hyperintensities were quantified by expert board-certified neurologist (GB), using the sum of the deep WM and periventricular scores of the Fazekas scale [29] (Fig. 1d). The separate deep WM and periventricular Fazekas sub-scores are reported in eTable 2.

#### DWI

Preprocessing included denoising, EPI-distortion and motion correction. WM microstructure was characterized using the Neurite Orientation Dispersion and Density Imaging (NODDI) [30, 31] with the intracellular volume fraction (V_ic_) and orientation dispersion index (ODI) values averaged over voxels belonging to the bilateral posterior limb of the internal capsule (PLIC) and cingulum bundle (CING), two WM regions consistently reported impaired in iNPH [14, 32] (Fig. 1e). The PLIC and CING were extracted based on the ICBM-DTI-81 atlas (Fig. 1b). NODDI models the local DWI signal as the sum of an intra-axonal compartment (V_ic_) with WM fibers showing a certain angular orientation dispersion (ODI), an extra-axonal, and an isotropic compartment, providing a finer-grain characterization of WM microstructure in clinical populations compared to tensor-based measures [33].

#### ASL

Preprocessing included EPI-distortion and motion correction. Relative perfusion in the bilateral thalami (THAL) and posterior cingulate cortices (PCC) was quantified by subtracting the labeled from the control ASL volume and normalizing the resulting value with respect to the average over all WM and GM voxels (Fig. 1f). Subcortical perfusion [34] and default mode network (DMN) function [15, 16] have been implicated in the pathophysiology of iNPH. The THAL was segmented using FreeSurfer6.0.0, and the PCC—the main DMN hub—was identified based on a fMRI-based segmentation [16] (Fig. 1b).

#### Rs-fMRI

Data were preprocessed and analyzed as previously described [16]. The DMN activity was characterized using a whole-brain co-activation pattern analysis with the PCC as seed region (Fig. 1g). This analysis identified three distinct DMN-related co-activation patterns encompassing the intra-network DMN functional connectivity (DMN_intra_), the functional connectivity between the DMN and lower order somatomotor and visual regions (DMN_SV_), and the functional connectivity between the DMN and higher order executive-control regions (DMN_EC_) [16]. DMN dynamics were quantified at the subject-level as the relative temporal occurrence of each network (DMN_intra_, DMN_SV_, DMN_EC_) [16].

### Statistical analysis

Comparisons between RSP and nRSP were performed using Student’s *t* test or ANCOVA including age as covariate (adding gender or education level as additional covariate did not change results) for normally distributed variables, Mann–Whitney *U* test for ordinal variables, and Chi-square test for categorical variables. Data normality was checked with Kolmogorov–Smirnov test. Bonferroni correction was applied for group-comparisons of 22 parameters of interest, thus setting the significance-level at *p* < 0.05/22. Effect size was quantified with Cohen’s *d* coefficient or η^2^ as appropriate. Moreover, post hoc power analyses setting *α* = 0.05 and power = 90% were performed.

Univariate prognostic value for CSFTT outcome of single parameters was assessed as the Area Under the Curve (AUC) of the receiver-operating characteristic (ROC) curve from logistic regressions with the group as dependent variable and the parameter of interest as independent variable. AUC 95% confidence intervals were estimated with bootstrapping (1000 bootstraps).

Multivariable prognostic values for CSFTT outcome of clinical (gait and neuropsychological) and neuroimaging (MRI) standardized variables were assessed with linear Support Vector Machine (lSVM) classifiers with leave-one-out cross-validation, and permutation testing for statistical significance assessment of out-of-sample accuracy, sensitivity, specificity, and AUC (1000 permutations). Missing data were imputed with the four-nearest-neighbor method.

Correlations between the parameters of interest were assessed with Spearman’s rank correlation.

Statistical analyses were performed using MatlabR2019b and G*Power3.1.

### Standard protocol approvals and patient consents

This study was approved by the ethical committee of Geneva University Hospitals (protocol NAC11-125). All subjects provided informed consent according to The Code of Ethics of the World Medical Association (Declaration of Helsinki).

## Results

### Participants and CSFTT response

In our iNPH cohort, 16 patients (53%) responded positively to CSFTT. Out of these 16 RSP, 2 improved in walking speed; 4 improved in TUG; 10 improved in both parameters (eFigure 1). Average absolute improvements of walking speed or TUG in RSP were 0.18 m/s and 6.1 s, respectively. 25 out of 30 iNPH patients had repeated walking speed assessment, with strong correlation between the two assessments (Pearson’s correlation: *r* = 0.96, *p* < 10^–13^ pre-CSFTT; *r* = 0.95, *p* < 10^–13^ post-CSFTT). Clinical features, beta-amyloid, phosphorylated and total tau levels were similar between RSP and nRSP (Table 1); this was unchanged when considering an alternative cutoff for responder definition (eTable1). INPH patients (both RSP and nRSP) were on average older, with a lower proportion of females, and lower education level than HCs. Out of the eight iNPH patients who underwent shunting, seven were CSFTT responders with average improvements of walking speed or TUG of 0.16 ms/s (27%) and 6.5 s (21%), respectively. One shunted patient was CSFTT non-responder, but experienced modest walking speed and TUG improvements of 0.06 ms/s (9%) and 0.2 s (1%). All shunted patients responded positively to surgery, as assessed with an inpatient visit at 6 weeks after surgery (improved gait and equilibrium were reported for all patients).

**Table 1:** Demographics and CSF biomarkers of iNPH patients responding and not responding to CSFTT, and healthy controls

Characteristics	iNPH RSP^a^ (*n* = 16)	iNPH nRSP^a^ (*n* = 14)	*p*-value^b^ RSP/nRSP	HC^a^ (*n* = 46)	*p*-value^c^ RSP/HC	*p*-value^c^ nRSP/HC
Age (years)	79.3 (6.6)	79.4 (5.2)	0.95	74.7 (5.4)	0.0081	0.0059
Gender, female (*n* (%))	6 (37%)	6 (43%)	0.76	35 (76%)	0.0050	0.019
Education level (I/II/III)—median [interq]	2 [1.0, 2.5]	1 [1.0, 1.0]	0.092	3 [2.0, 3.0]	0.0069	0.000065
Aβ_1-42_ (ng/l)	757.9 (282.5)	677.0 (231.5)	0.40	–	–	–
pTau (ng/l)	45.8 (14.6)	42.6 (12.8)	0.54	–	–	–
tTau (ng/l)	242.1 (113.9)	236.6 (93.4)	0.89	–	–	–

*iNPH* idiopathic normal pressure hydrocephalus, *RSP* responder, *nRSP* non-responder, *HC* healthy control, *interq* interquartile range, *Aβ*_*1-42*_ 42 amino-acid form of beta-amyloid, *pTau* phosphorylate tau, *tTau* total tau

^a^Group-level mean (standard deviation), median [25th–75th interquartile range] or number of subjects (percentage) per class are reported as appropriate

^b^Student’s *t* test, Chi-square (gender) or Mann–Whitney *U* test (education level) were used as appropriate

^c^Generalized linear model including age as covariate. Results were unchanged when adding gender or education level as covariates. Age, gender and education level were compared using Student’s *t* test, Chi-square and Mann–Whitney *U* test, respectively

### Differences between iNPH patients and controls

All gait and neuropsychological parameters were impaired in both RSP and nRSP compared to HCs, except for the step width which was impaired in nRSP only (Table 2; eTable1). Concerning the neuroimaging parameters, both RSP and nRSP had larger ventricles than HCs, consistently with the diagnostic definition of iNPH; decreased posterior cingulate sulcal volume; increased calcarine fissure volume (Table 2; eTable1). Both RSP and nRSP had lower orientation dispersion of periventricular WM fibers than HCs, suggesting compression of these WM bundles but no major axonal loss since the intra-axonal volume fraction (V_ic_) was unaffected [30, 33] (Table 2; eTable1). Both RSP and nRSP had stronger functional connectivity between the DMN and executive-control regions (DMN_EC_), while RSP only had lower functional connectivity within the DMN (DMN_intra_) compared to HCs. Finally, WM lesion load was higher in nRSP only compared to HCs [results were similar when considering an alternative cutoff for responder definition (eTable1) or when considering separately the deep and periventricular WM sub-scores (eTable 3)].

**Table 2:** Clinical and imaging characteristics of iNPH patients responding and not responding to CSFTT and healthy controls

Characteristics	iNPH RSP^a^ (*n* = 16)	iNPH nRSP^a^ (*n* = 14)	*p*-value (effect size)^b^ RSP/nRSP	AUC^c^ RSP/nRSP	N_total_^d^ (α 0.05, power 90%)	HC^a^ (*n* = 46)	*p*-value^e^ RSP/HC	*p*-value^e^ nRSP/HC
Disease duration (months)	31.1 (20.4)	26.6 (16.3)	0.63 (− 0.24)	0.54 [0.33, 0.74]	736	–	–	–
Walking speed (m/s)	0.71 (0.26)	0.83 (0.26)	0.21 (0.47)	0.63 [0.41, 0.83]	194	1.23 (0.13)	< 10^–12^**	< 10^–8^**
Step length (m)	0.83 (0.27)	0.97 (0.26)	0.16 (0.53)	0.68 [0.42, 0.85]	154	1.31 (0.10)	< 10^–12^**	< 10^–7^**
Step width (m)	0.09 (0.02)	0.11 (0.03)	0.069 (0.69)	0.68 [0.44, 0.85]	92	0.07 (0.02)	0.011	0.00012**
TUG (s)	22.9 (12.3)	18.6 (8.3)	0.28 (− 0.40)	0.61 [0.35, 0.78]	266	10.6 (2.0)	< 10^–6^**	< 10^–5^**
Categorical verbal fluency (*n*)	12.88 [4.485]	11.36 [4.534]	0.37 (− 0.34)	0.61 [0.36, 0.78]	342	19.91 [4.652]	.000033**	< 10^–6^**
WAIS-III symbol digit modalities (*n*)^f^	31.3 (10.1)	24.9 (13.7)	.20 (− 0.54)	0.61 [0.39, 0.79]	146	54.3 (15.8)	< 10^–5^**	< 10^–6^**
FCSRT immediate free recall (*n*)^g^	14.9 (7.8)	13.8 (8.1)	0.73 (− 0.14)	0.54 [0.26, 0.73]	2156	26.4 (7.1)	0.000017**	0.000037**
Starkstein apathy scores (*n*)^h^	15.5 (6.2)	14.8 (5.4)	0.76 (− 0.12)	0.56 [0.38, 0.79]	2934	9.5 (3.2)	0.000053**	0.00029**
Relative ventricle volume (%)	3.82 (0.87)	3.59 (0.73)	0.44 (− 0.29)	0.56 [0.34, 0.77]	472	1.26 (0.49)	< 10^–18^**	< 10^–17^**
Relative PCMF volume (%)	0.090 (0.041)	0.070 (0.052)	0.24 (− 0.43)	0.70 [0.45, 0.89]	216	0.13 (0.048)	0.00037**	0.000018**
Relative CF volume (%)	0.206 (0.056)	0.207 (0.061)	0.93 (0.03)	0.51 [0.29, 0.71]	> 10,000	0.115 (0.031)	< 10^–8^**	< 10^–7^**
ODI-PLIC^i^	0.094 (0.018)	0.087 (0.011)	0.25 (− 0.45)	0.61 [0.40, 0.79]	212	0.131 (0.024)	< 10^–5^**	< 10^–6^**
Vic-PLIC^i^	0.768 (0.084)	0.758 (0.078)	0.74 (− 0.13)	0.58 [0.38, 0.77]	2500	0.770 (0.076)	0.98	0.67
ODI-CING^i^	0.207 (0.090)	0.158 (0.074)	0.14 (− 0.59)	0.69 [0.46, 0.86]	124	0.302 (0.085)	0.000045**	< 10^–6^**
Vic-CING^i^	0.531 (0.074)	0.550 (0.076)	0.50 (0.26)	0.58 [0.39, 0.78]	628	0.516 (0.067)	0.17	0.090
DMN_intra_	0.24 (0.11)	0.29 (0.12)	0.31 (0.38)	0.61 [0.40, 0.81]	296	0.40 (0.13)	0.00028**	0.0061
DMN_SV_	0.25 (0.11)	0.22 (0.12)	0.44 (− 0.29)	0.58 [0.31, 0.76]	504	0.25 (0.10)	0.95	0.35
DMN_EC_	0.50 (0.13)	0.49 (0.15)	0.84 (− 0.08)	0.53 [0.33, 0.77]	6′168	0.35 (0.11)	0.00011**	0.00027**
Perf THAL^j^	1.44 (0.94)	1.44 (0.43)	0.99 (0.01)	0.48 [0.27, 0.69]	> 10′000	1.41 (0.35)	0.60	0.74
Perf PCC^j^	0.98 (0.37)	0.98 (0.58)	0.99 (0.01)	0.51 [0.29, 0.73]	> 10′000	1.40 (0.41)	0.0078	0.016
Fazekas—median [interq]	3 [1.5, 5.0]	4 [4.0, 6.0]	0.030 (0.83)	0.69 [0.43, 0.82]	62	2 [2.0, 4.0]	0.61	0.0013**

*iNPH* idiopathic normal pressure hydrocephalus, *RSP* responder, *nRSP* non-responder, *AUC* area under the curve, *HC* healthy control, *TUG* timed up and go test, *WAIS-III* Wechsler Adult Intelligence Scale symbol digit modalities test, *FCSRT* French version of the Free and Cued Selective Reminding Test

^a^Group-level mean (standard deviation), median [25th–75th interquartile range] or number of subjects per class is reported as appropriate

^b^Student’s *t* test, Chi-square (gender) or Mann–Whitney *U* test (education level) was used as appropriate. Cohen’s *d* or η^2^ effect sizes are reported as appropriate in parenthesis (positive values indicate largest mean (median) in the nRSP group compared to the RSP group). **p < 0.0023 (surviving Bonferroni correction for 22 comparisons)

^c^AUC from univariate RSP/nRSP logistic regression. 95% confidence intervals are reported in square brackets

^d^Post hoc power analysis with alpha set at 0.05 and power set at 90%. N_total_ = total number of patients that would be needed to reach statistical significance

^e^Generalized linear model including age as covariate. Results were unchanged when adding gender or education level as covariates. Age, gender and education level were compared using Student’s *t* test, Chi-square and Mann–Whitney *U* test, respectively. **p <0.0024 (surviving Bonferroni correction for 21 comparisons)

^f^WAIS-III symbol digit modalities score was missing for 6 iNPH patients (2 RSP, 4 nRSP)

^g^FCSRT immediate free recall score was missing for 3 iNPH patients (2 RSP, 1 nRSP) and 1 HC

^h^Starkstein apathy score was missing for 2 iNPH patients (1 RSP, 1 nRSP)

^i^DWI data were missing for 2 iNPH patients (nRSP)

^j^ASL data were missing for 2 iNPH patients (nRSP) and 1 HC

### Univariate prediction of CSFTT outcome

**Fig. 2:**
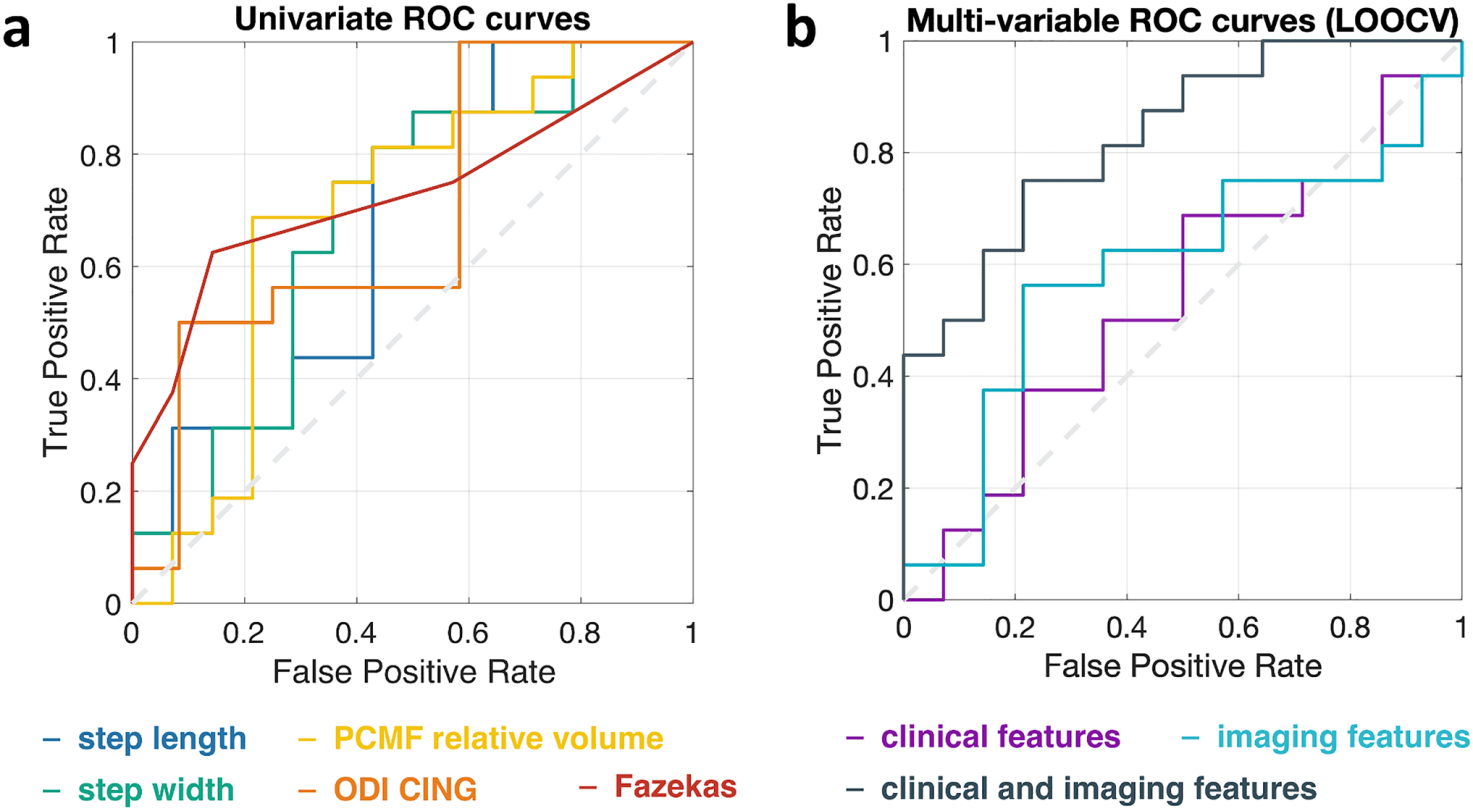
(**a**) ROC curves obtained from logistic regression (univariate CSFTT outcome prediction) with best performing parameters: step length, step width, posterior cingulate fissure morphology (PCMF relative volume), cingulum WM microstructure (ODI-CING), WM lesion load (Fazekas score). Univariate ROC curves were drawn considering the whole dataset and not within a cross-validation setting. (**b**) ROC curves obtained from three distinct lSVM multivariable classifiers, including clinical, imaging, and clinical plus imaging features, and leave-one-out cross-validation. *PCMF *posterior callosal marginal fissure, *ODI*, orientation dispersion index, *CING*, cingulum bundle, *LOOCV*, leave-one-out cross-validation

In accordance with the RSP-nRSP group-comparisons, AUC values from logistic regressions indicated low (chance-level) univariate prognostic accuracy for CSFTT outcome for all parameters (all 95% confidence intervals included 0.5 chance-level value, Table 2, eTable 1). The features with the highest univariate AUC were gait (step length AUC = 0.68 [0.43–0.86]; step width AUC = 0.68 [0.45–0.87]), posterior cingulate sulcal volume (PCMF volume AUC = 0.70 [0.48–0.86]), cingulum WM microstructure (ODI-CING AUC = 0.65 [0.43–0.83]) and WM lesion load (Fazekas AUC = 0.69 [0.45–0.81]) (Fig. 2a; Table 2; eTable 1). The absence of significant linear relationships between relative changes in walking speed or TUG after CSFTT, and any of the gait, neuropsychological and neuroimaging parameters further indicates that results were not driven by the particular choice of 10% improvement used to define the RSP and nRSP groups (eTable 4, eTable 5).

Post hoc power analysis shows the large number *N*_total_ of patients that would be needed to reach univariate statistical significance for the parameters of interest, with only the step width and WM lesion load having *N*_total_ < 100 (Table 2).

### Multivariable prediction of CSFTT outcome

Three lSVM classifiers were trained on 9 clinical (disease duration, walking speed, step length, step width, TUG, categorical verbal fluency, FCSRT, WAISS-III, Starkstein, plus education level and age); 13 imaging (ventricle, PCMF, CF relative volumes, ODI-PLIC, V_ic_-PLIC, ODI-CING, V_ic_-CING, DMN_intra_, DMN_SV_, DMN_EC_, THAL-perfusion, PCC-perfusion, Fazekas score); all the 22 parameters (plus education level and age), respectively. The pair-wise correlations between the considered parameters, in the whole iNPH group and in RSP and nRSP separately, are reported in eFigure 2 and mainly highlight intra-domain relationships (i.e., correlations between ODI and V_ic_; between walking speed, step length, and TUG; between step width and TUG in nRSP only).

**Fig. 3:**
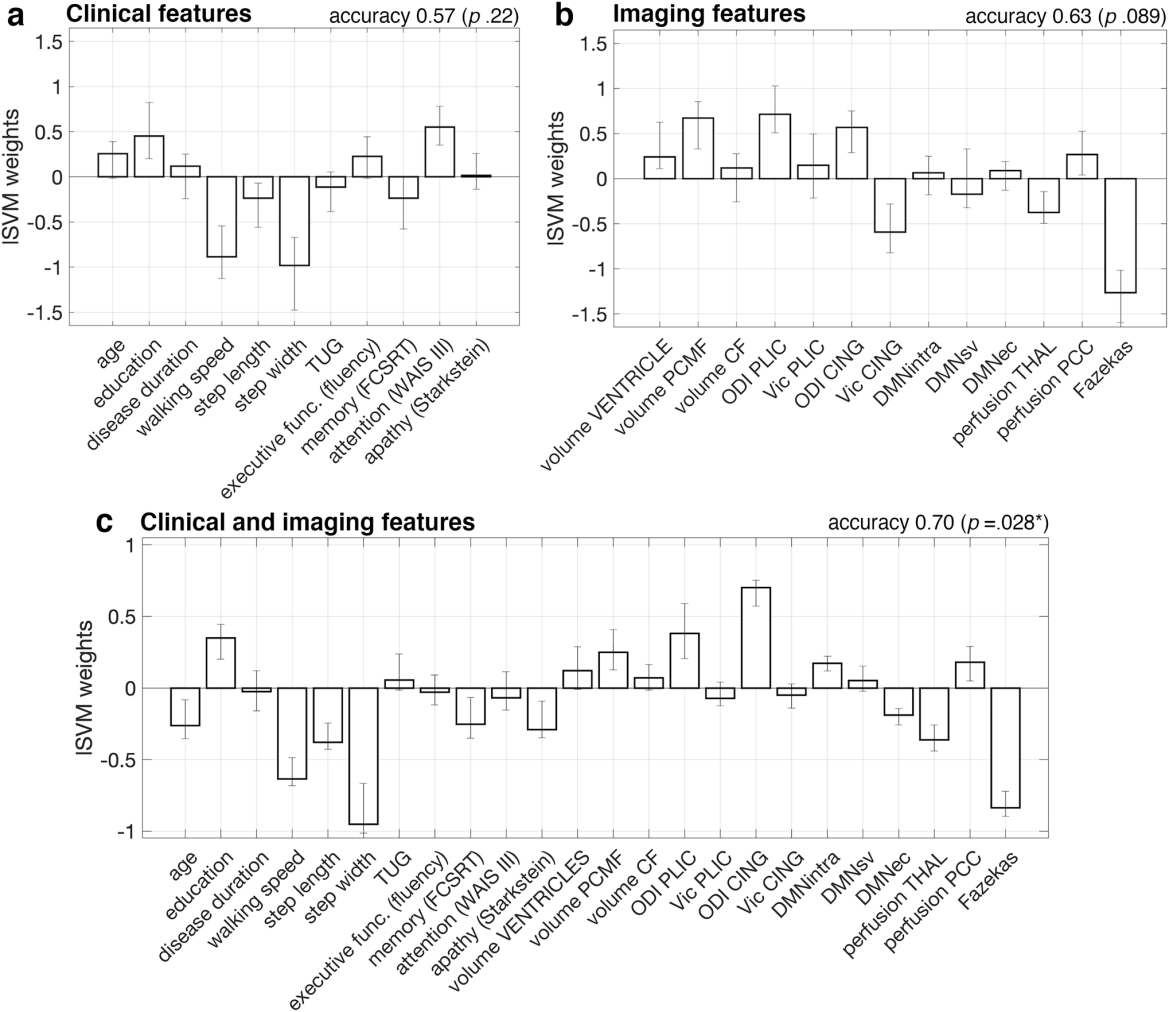
Feature weights obtained from three lSVM classifiers trained on 9 clinical features plus age and education level (**a**), 13 imaging features (**b**), or both clinical and imaging features (**c**). Out-of-sample accuracy from leave-one-out cross-validation and bootstrap *p*-values are reported above each plot (*p < 0.05). Bars indicate average weights, and 5–95 percentiles of the weight distributions estimated over 30 leave-one-out cross-validation loops. *lSVM*, linear support vector machine classifies, *TUG*, timed up and go test, *executive func.*,  executive functions, *FCSRT*, French version of the Free and Cued Selective Reminding Test immediate free recall test, *WAIS III* Wechsler Adult Intelligence Scale symbol digit modalities test, *PCMF*  posterior callosal marginal fissure, *CF* calcarine fissure, *ODI *orientation dispersion index, *Vic* intracellular volume fraction, *PLIC* posterior limb of the internal capsule, *CING *cingulum bundle, *DMNintra *functional connectivity between default mode network (DMN) regions, *DMNsv* functional connectivity between DMN and somatomotor and visual regions, *DMNec* functional connectivity between DMN and executive-control regions, *THAL *thalamus, *PCC* posterior cingulate cortex

Cross-validation classification accuracy was above chance-level for the combined clinical + imaging classifier only [clinical: out-of-sample accuracy/sensitivity/specificity/AUC = 0.57/0.63/0.50/0.54 (*p* = 0.22/0.15/0.25/0.38); imaging: out-of-sample accuracy/sensitivity/specificity/AUC = 0.63/0.56/0.71/0.59 (*p* = 0.089/0.28/0.021/0.21); clinical + imaging: out-of-sample accuracy/sensitivity/specificity/AUC = 0.70/0.75/0.64/0.83 (*p* = 0.028/0.022/0.051/0.0010)]. The lSVM weights indicate which parameters are most relevant to the prediction task and suggest that, in a multivariable prediction setting, positive CSFTT outcome is associated with slower walking speed and smaller step width at baseline, larger orientation dispersion of periventricular WM fibers (ODI-CING), and lower WM lesion load (Fig. 3). The AUC and 95% confidence intervals of the three classifiers with respect to the whole dataset (i.e., irrespectively of cross-validation) were 0.85 [0.65–0.95], 0.88 [0.66–0.98], and 1 [0.99–1], respectively (Fig. 2b).

## Discussion

The CSFTT has high positive predictive value for surgery outcome and, despite its invasive nature, is used in several iNPH centers as prognostic test [7, 8]. This study supports the usage of CSFTT in the clinical management of iNPH by showing that its outcome cannot be easily predicted by a single gait, neuropsychological or neuroimaging parameters. However, integrating clinical and imaging parameters obtained from non-invasive patient assessments helps identifying patients who will likely respond to CSFTT. In such a multivariable setting, we show that gait parameters, WM lesions and periventricular WM fiber organization contribute the most to symptom reversibility prediction, while cognition and brain function contribute the least. Yet, the modest prediction accuracy that can be achieved by combining these factors do not stand for replacing the standard CSFTT procedure.

Gait impairment is the hallmark of iNPH, with patients presenting different gait and balance alterations [12] often including wide-based and shuffling gait with step shortening [35]. Our results indicate that a gait phenotype with normal step width but slow gait and short step length tends to have better CSFTT outcome than a phenotype with wide-based gait (suggesting poor balance control) and relatively preserved walking speed. Yet, slow gait was observed in both RSP and nRSP but could have different origins in the two patients’ subgroups. Reduced walking speed was associated with wider steps in nRSP only [nRSP: ρ_walking speed, step width_ = − 0.83 (*p* < 10^–3^); RSP: ρ_walking speed, step width_ = − 0.03 (*p* = 0.91); eFigure 2], pinpointing a specific nRSP phenotype with interrelated dynamic unbalance and slow gait. The TUG, another indicator of dynamic balance, did not contribute to RSP/nRSP prediction but positively correlated with step width in nRSP only [nRSP: ρ_TUG, step width_ = 0.74 (*p* = 0.0033); RSP: ρ_TUG, step width_ = 0.07 (*p* = 0.80); eFigure 2]. These results are in line with previous studies indicating that balance-related gait parameters do not improve after CSFTT [16, 23] and patients with moderate-to-severe postural instability do not show long-term improvement after shunting [36]. However, recent findings on younger iNPH patients show improved dynamic equilibrium after shunting, suggesting that a patient stratification based on age and disease duration may provide a better characterization of symptom reversibility [37]. Moreover, the reasons why poor balance may not predict CSFTT outcome are unclear. One hypothesis is that balance control may be specifically bounded to brain circuits suffering from irreversible brain damage related to ventriculomegaly [23]. Yet, overlaps between balance and gait circuits, and neural substrates of different gait phenotypes should be further investigated.

Brain imaging features significantly contributed to RSP/nRSP discrimination and demonstrated a moderate-to-good negative predictive value for CSFTT outcome (lSVM imaging classifier specificity = 0.71, *p* = 0.021). WM lesions and microstructure of the cingulum bundle contributed the most to prediction. Hyperintensities in the T2w MRI contrast are unspecific markers of WM damage, associated with small vessel disease in older populations, but also with focal edema due to dysfunctional transependymal transportation in iNPH [38]. The spatial distribution of WM lesions can be informative of underlying pathophysiological processes, with periventricular but not deep WM lesions being reduced by acetazolamide treatment in iNPH patients [38]. In this study, WM lesion load was quantified with the total Fazekas scale which combines both periventricular and deep WM contributions [29]. The periventricular and deep WM lesion contributions were equal in nRSP, suggesting a shared pathophysiological substrate, but unbalanced in RSP, suggesting multiple pathophysiological substrates (eTable 3). One hypothesis is that WM lesions in nRSP relate to non-reversible cerebrovascular factors, thus hindering a positive response to CSFTT, while WM lesions in RSP partly relate to reversible iNPH mechanisms, such as transependymal edema, possibly relieved by CSFTT.

Low orientation dispersion of periventricular WM fibers was also associated with poor CSFTT outcome in the multivariable analyses. An ODI decrease indicates abnormal hyper-alignment of WM fibers, possibly caused by compression and stretching of the WM bundles [30]. A previous study reported decreased ODI in iNPH compared to HCs in the periventricular section of the corticospinal tract [33] and the finding is here extended to the cingulum bundle. In addition, the lower ODI observed in nRSP compared to RSP suggests that a more important stretching of the cingulum and, to a less extent, of the posterior limb of the internal capsule preclude gait improvement after CSFTT. Nonetheless, there was no association between periventricular ODI and ventricles volume (eFigure 2), and the latter did not predict CSFTT outcome, which complicates the link between ventriculomegaly and mechanical/deformation effects onto the WM. Changes of the subarachnoid space may also represent a stressor onto brain tissues and have treatment implications [27]. In our sample, the posterior cingulate and calcarine fissures were, respectively, constrained and enlarged in iNPH compared to HCs, consistently with previous findings [28]. In the multivariable prediction analyses, less constrained posterior cingulate fissure (i.e., more pronounced high-convexity tightness), together with stronger hyper-alignment of cingular WM fibers, were associated with poor CSFTT outcome. It might be that the removal of 40 ml CSF is not enough to produce brain changes and short-term symptom reversal in patients with more pronounced morphological and microstructural brain alterations, which may not preclude future response to shunting. Finally, although to our knowledge this is the first study investigating the relationship between NODDI parameters and short-term symptom reversibility, others have reported an association between fractional anisotropy and axial diffusivity in the corticospinal tract and symptom improvement after shunting [32]. These diffusion tensor parameters are unspecific markers of WM microstructure and can represent a mixture of WM deformation and neurodegeneration [33]. In our sample, there was no alteration of intracellular volume fraction in patients compared to HCs, suggesting limited neurodegeneration.

Among the imaging features, the functional ones (brain perfusion and functional connectivity) contributed the least to CSFTT outcome prediction. Previous findings on the predictive utility of cerebral perfusion are discordant: one study found an association between higher baseline perfusion in medial-frontal cortex and shunt response [39], but another study did not find any association [40]. In our sample, perfusion in the posterior cingulate cortex and thalamus did not predict CSFTT outcome, but it was on average lower in iNPH compared to HCs. Alterations of cerebral perfusion can have different pathophysiological substrates. Transependymal edema in periventricular brain tissues may lead to compression of small vessels and reduced elimination of vasoactive metabolites [34], a process that could be partially reverted with CSF removal. However, reduced perfusion may also be linked to vascular risk factors (prevalent among iNPH patients [41]) and, therefore, be unrelated to iNPH reversibility mechanisms.

Changes of DMN functional dynamics have been implicated in the pathophysiology of iNPH [15, 16] and are partially reverted by CSFTT [16]. Yet, we found no association between baseline DMN dynamics and CSFTT response. Functional neuroimaging modalities may be sensitive to short-term functional plasticity mechanisms occurring even few hours after CSF removal, but these changes may not be directly associated with short-term clinical changes.

Finally, cognition and education level did not predict CSFTT outcome. However, patients included in this study had long disease duration (29 months on average) preventing generalization for patients with shorter disease durations [42]. Cognitive impairments tend to improve less than gait after CSFTT or shunting [18] and may partially result from non-reversible iNPH pathophysiological processes (e.g., secondary neurodegeneration) or alternate pathways (e.g., AD). Yet, in our study RSP and nRSP did not differ in AD biomarkers, suggesting a dissociation between Alzheimer’s pathology and iNPH symptom reversibility.

The strengths of this study include the availability of multimodal MR brain imaging and quantitative gait assessment in iNPH patients before and after CSFTT. However, the CSFTT has poor specificity for shunting outcome prediction [43, 44], so that a subset of our nRSP patients may still experience symptom improvement after shunting. Only 8 out of the 30 patients included in this study underwent shunting, with positive outcome at 6-week ambulatorial follow-up. The eight shunted patients were CSFTT responders (seven patients) or experienced moderate post-CSFTT gait improvement (one patient), indicating that in our Center only patients who experience moderate-to-good CSFTT response are referred for surgery. The limited sample size of the shunted groups, the absence of shunted patients with negative outcome at 6 weeks, and the lack of longer post-surgical follow-up precluded an analysis of shunt-response prediction in relation to baseline multimodal parameters and CSFTT response. This study was based on an educated choice of brain regions and features of interest. This was necessary to achieve a trade-off between problem complexity (number of investigated variables) and sample size. CSFTT-related effects outside the considered regions of interest may be present. Finally, the definition of CSFTT responder was based on a percentage cutoff on walking speed and TUG. Although group-comparisons with an alternative cutoff, and correlations between gait changes and variables of interest suggest that our results are not driven by this particular definition, the quantification of clinical improvement after CSFTT remains a matter of debate [45]. Future studies may attempt to use clinical and neuroimaging parameters to predict CSFTT response along multiple clinimetric axes.

## Conclusions

To conclude, our negative results show that single clinical or neuroimaging parameters do not predict CSFTT outcome, indirectly supporting its utility as prognostic tool. Multivariable classification analyses highlight the value of combining clinical and imaging features to achieve robust, although moderate prediction accuracy of CSFTT outcome which, however, does not stand for replacing the standard CSFTT procedure. RSP classification sensitivity and specificity were, respectively, 75% and 64%, indicating that gait and WM parameters together can help identifying patients more likely to experience short-term symptom reversibility but cannot exclude patients from further CSFTT. These results strongly encourage future investigations on the multivariable predictive value of gait and WM features for shunt surgery outcome.

## Supplementary Information

Below is the link to the electronic supplementary material.

Supplementary file1 (DOCX 608 KB)
