# Supplementary material for: CSF tap test in idiopathic normal pressure hydrocephalus: still a necessary prognostic test?
Source: J Neurol. 2022 May 22;269(9):5114–26. doi: 10.1007/s00415-022-11168-x (PMC9363476; doi:10.1007/s00415-022-11168-x)

**CSF tap test in idiopathic Normal Pressure Hydrocephalus: Still a necessary prognostic test?**

Alessandra Griffa^1,2,3^, PhD, Giulia Bommarito^4^, MD PhD, Frédéric Assal^4^, MD, Maria Giulia Preti^2,5,1^, PhD, Rachel Goldstein^4^, Stéphane Armand^6^, PhD, François R. Herrmann^7^, MD PhD, Dimitri Van De Ville^2,1^, PhD, Gilles Allali^3,4,9^, MD PhD

1. Department of Radiology and Medical Informatics, University of Geneva (UNIGE), Geneva, Switzerland

2. Institute of Bioengineering, Center of Neuroprosthetics, Ecole Polytechnique Fédérale De Lausanne (EPFL), Geneva, Switzerland

3. Leenaards Memory Center, Lausanne University Hospital and University of Lausanne, Lausanne, Switzerland.

4. Department of Clinical Neurosciences, Division of Neurology, Geneva University Hospitals and Faculty of Medicine, University of Geneva, Geneva, Switzerland

5. CIBM Center for Biomedical Imaging, Switzerland

6. Kinesiology Laboratory, Geneva University Hospitals and Faculty of Medicine, University of Geneva, Geneva, Switzerland

7. Department of Rehabilitation and Geriatrics, Geneva University Hospitals and University of Geneva, Geneva, Switzerland

8. Department of Radiology and Medical Informatics, University of Geneva, Geneva, Switzerland

9. Department of Neurology, Division of Cognitive & Motor Aging, Albert Einstein College of Medicine, Yeshiva University, Bronx, NY, USA

**SUPPLEMENTARY INFORMATION**

**eTable1**. **Sensitivity analysis with respect to the CSFTT responders’ definition**

|  | iNPH RSP^(a)^ ≥ 15%  (n = 13) | iNPH nRSP^(a)^ < 15%  (n = 17) | *p*-value (effect size)^(b)^  RSP / nRSP | AUC^(c)^  RSP / nRSP | *p*-value^(d)^  RSP / HC | *p*-value^(d)^  nRSP / HC |
| --- | --- | --- | --- | --- | --- | --- |
| DEMOGRAPHICS AND CSF BIOMARKERS | | | | | | |
| Age (years) | 79.6 (6.4) | 79.2 (5.6) | .84 | - | .0080 | .0061 |
| Gender, female (n (%)) | 5 (38%) | 7 (41%) | .88 | - | .010 | .0091 |
| Education level (I / II / III) – median [interq] | 2 [1.0, 2.3] | 1 [1.0, 1.3] | .090 | - | .013 | .000052 |
| A𝛽_1-42_ (ng/l) | 741.7 (304.4) | 703.7 (225.3) | .70 | - | - | - |
| pTau (ng/l) | 45.9 (14.6) | 43.1 (13.1) | .59 | - | - | - |
| tTau (ng/l) | 246.8 (117.3) | 233.9 (94.2) | .74 | - | - | - |
| CLINICAL AND IMAGING CHARACTERISTICS | | | | | | |
| Disease duration (months) | 31.8 (19.8) | 26.8 (17.6) | .54 (-0.27) | 0.55 [0.30, 0.78] | - | - |
| Walking speed (m/s) | 0.71 (0.27) | 0.81 (0.26) | .30 (0.39) | 0.60 [0.34, 0.78] | <10^-13^** | <10^-11^** |
| Step length (m) | 0.83 (0.28) | 0.94 (0.26) | .25 (0.43) | 0.65 [0.40, 0.82] | <10^-13^** | <10^-10^** |
| Step width (m) | 0.09 (0.03) | 0.11 (0.02) | .043 (0.78) | 0.71 [0.47, 0.86] | .031 | .0000017** |
| TUG (s) | 23.4 (13.3) | 18.9 (8.2) | .26 (-0.42) | 0.60 [0.38, 0.79] | <10^-7^** | <10^-7^** |
| Categorical verbal fluency (n) | 12.8 (3.2) | 11.7 (5.3) | .53 (-0.23) | 0.60 [0.38, 0.80] | <10^-5^** | <10^-6^** |
| WAIS-III symbol digit modalities (n)^(e)^ | 31.0 (11.0) | 26.7 (12.8) | .40 (-0.35) | 0.57 [0.37, 0.79] | .000023** | <10^-6^** |
| FCSRT immediate free recall (n) ^(f)^ | 16.1 (8.4) | 13.2 (7.4) | .36 (-0.36) | 0.63 [0.40, 0.81] | .00011** | <10^-7^** |
| Starkstein apathy scores (n) ^(g)^ | 15.5 (5.7) | 15.0 (6.0) | .83 (-0.09) | 0.58 [0.36, 0.78] | .000012** | .000024** |
| Relative ventricle volume (%) | 3.82 (0.94) | 3.62 (0.70) | .50 (-0.25) | 0.56 [0.34, 0.77] | <10^-18^** | <10^-21^** |
| Relative PCMF volume (%) | 0.095 (0.044) | 0.071 (0.047) | .17 (-0.51) | 0.69 [0.48, 0.87] | .030 | .000098** |
| Relative CF volume (%) | 0.203 (0.059) | 0.209 (0.057) | .77 (0.11) | 0.55 [0.36, 0.76] | <10^-8^** | <10^-10^** |
| ODI PLIC^(h)^ | 0.091 (0.018) | 0.091 (0.014) | .95 (0.02) | 0.48 [0.26, 0.72] | <10^-6^** | <10^-8^** |
| Vic PLIC^(h)^ | 0.760 (0.083) | 0.767 (0.079) | .81 (0.09) | 0.51 [0.33, 0.74] | .68 | .91 |
| ODI CING^(h)^ | 0.204 (0.091) | 0.171 (0.081) | .32 (-0.39) | 0.64 [0.40, 0.82] | .00062** | .0000022** |
| Vic CING^(h)^ | 0.522 (0.056) | 0.554 (0.085) | .26 (0.43) | 0.59 [0.38, 0.79] | .75 | .078 |
| DMN_intra_ | 0.239 (0.123) | 0.285 (0.108) | .28 (0.41) | 0.64 [0.37, 0.83] | .00021** | .0021** |
| DMN_SV_ | 0.264 (0.110) | 0.215 (0.116) | .25 (-0.43) | 0.62 [0.38, 0.79] | .62 | .30 |
| DMN_EC_ | 0.498 (0.131) | 0.500 (0.143) | .97 (0.02) | 0.52 [0.30, 0.74] | .00021** | .000068** |
| Perf THAL^(i)^ | 1.237 (0.526) | 1.599 (0.868) | .20 (0.49) | 0.57 [0.35, 0.78] | .18 | .22 |
| Perf PCC^(i)^ | 0.987 (0.394) | 0.975 (0.527) | .95 (-0.02) | 0.52 [0.31, 0.73] | .0021** | .0020** |
| Fazekas – median [interq] | 3 [1.7 6] | 4 [3.7, 6.0] | .15 (0.52) | 0.61 [0.38, 0.78] | .41 | .0012** |

Abbreviations: iNPH = idiopathic Normal Pressure Hydrocephalus; RSP = responder; nRSP = non-responder; HC = healthy control; interq = interquartile range; A𝛽_1-42_ = 42 amino-acid form of beta-amyloid; pTau = phosphorylate tau; tTau = total tau.

^(a)^ Group-level mean (standard deviation), median [25^th^-75^th^ interquartile range], or number of subjects (percentage) per class are reported as appropriate.

^(b)^ Student’s t-test, chi-square (gender) or Mann-Whitney u-test (education level; Fazekas score) were used as appropriate. Cohen’s *d* or 𝜂^2^ effect sizes are reported as appropriate in parenthesis (positive values indicate largest mean (median) in the nRSP group compared to the RSP group). **: *p* < .0023 (surviving Bonferroni correction for 22 comparisons).

^(c)^ AUC from univariate RSP/nRSP logistic regression. 95% confidence intervals are reported in square brackets.

^(d)^ Generalized linear model including age as covariate. Results were unchanged when adding gender or education level as covariates. Age, gender and education level were compared using Student’s t-test, chi-square and Mann-Whitney u-test, respectively. **: *p* < .0024 (surviving Bonferroni correction for 21 comparisons).

^(e)^ WAIS-III symbol digit modalities score was missing for 6 iNPH patients (2 RSP, 4 nRSP).

^(f)^ FCSRT immediate free recall score was missing for 3 iNPH patients (2 RSP, 1 nRSP) and 1 HC.

^(g)^ Starkstein apathy score was missing for 2 iNPH patients (1 RSP, 1 nRSP).

^(h)^ DWI data were missing for 2 iNPH patients (nRSP).

^(i)^ ASL data were missing for 2 iNPH patients (nRSP) and 1 HC

**eTable2**. **Multimodal MR brain imaging: sequence parameters**

| MRI sequence | Voxel size (mm) | TR^a^ (ms) | TE^b^ (ms) | Second TE (ms) | Number of acquired volumes | Number of b0 volumes | Number of shells | b-value (s/mm^2^) |
| --- | --- | --- | --- | --- | --- | --- | --- | --- |
| T1-weighted (T1w) | 0.8 x 0.8 x 0.8 | 2200 | 2.4 | - | 1 | - | - | - |
| T2-weighted (T2w) | 0.4 x 0.4 x 4.0 | 4000 | 110 | - | 1 | - | - | - |
| Diffusion Weighted Imaging (DWI) | 1.8 x 1.8 x 1.8 | 4400 | 76 | - | 94 | 4 | 3 | 200 / 1000 / 4000 |
| Arterial Spin Labeling (ASL) | 3.0 x 3.0 x 3.0 | 5000 | 16.18 | - | 2 | - | - | - |
| Resting-state functional imaging (rs-fMRI) | 2.5 x 2.5 x 2.5 | 1057 | 30 | - | 600 | - | - | - |
| Field map^c^ | 2.5 x 2.5 x 2.5 | 600 | 4.49 | 6.95 | 2 | - | - | - |

^(a)^ Repetition time

^(b)^ Echo time

^(c)^ Field map: two field map volumes with reversed phase encoding directions were acquired for susceptibility distortion correction

**eTable3**. **Sub-components of Fazekas scores for iNPH patients responding and not responding to CSFTT and group comparisons**

| RSP patients | Fazekas [0 - 6] | Fazekas_DWM_ [0 - 3]^(a)^ | Fazekas_PV_ [0 - 3]^(a)^ | ( Fazekas_DWM –_  Fazekas_PV_ )^(b)^ |  | nRSP patients | Fazekas [0 - 6] | Fazekas_DWM_ [0 - 3]^(a)^ | Fazekas_PV_ [0 - 3]^(a)^ | ( Fazekas_DWM –_  Fazekas_PV_ )^(c)^ |  | Comparison RSP / nRSP | *p*-value (effect size)^(d)^ |
| --- | --- | --- | --- | --- | --- | --- | --- | --- | --- | --- | --- | --- | --- |
| 1 | 3 | 1 | 2 | -1 |  | **1** | 6 | 3 | 3 | 0 |  | **Fazekas** | .030 (0.15) |
| 2 | 3 | 1 | 2 | -1 |  | **2** | 4 | 2 | 2 | 0 |  | **Fazekas_DWM_** | .035 (0.13) |
| 3 | 6 | 3 | 3 | 0 |  | **3** | 4 | 2 | 2 | 0 |  | **Fazekas_PV_** | .080 (0.09) |
| 4 | 3 | 1 | 2 | -1 |  | **4** | 6 | 3 | 3 | 0 |  |  |  |
| 5 | 6 | 3 | 3 | 0 |  | **5** | 6 | 3 | 3 | 0 |  |  |  |
| 6 | 1 | 1 | 0 | 1 |  | **6** | 2 | 1 | 1 | 0 |  | **Comparison RSP / HC** | ***p*-value ^(e)^** |
| 7 | 1 | 1 | 0 | 1 |  | **7** | 4 | 2 | 2 | 0 |  | **Fazekas** | .62 |
| 8 | 1 | 1 | 0 | 1 |  | **8** | 4 | 2 | 2 | 0 |  | **Fazekas_DWM_** | .41 |
| 9 | 2 | 1 | 1 | 0 |  | **9** | 4 | 2 | 2 | 0 |  | **Fazekas_PV_** | .87 |
| 10 | 6 | 3 | 3 | 0 |  | **10** | 6 | 3 | 3 | 0 |  |  |  |
| 11 | 6 | 3 | 3 | 0 |  | **11** | 4 | 2 | 2 | 0 |  |  |  |
| 12 | 4 | 2 | 2 | 0 |  | **12** | 3 | 1 | 2 | -1 |  | **Comparison nRSP / HC** | ***p*-value ^(e)^** |
| 13 | 3 | 1 | 2 | -1 |  | **13** | 6 | 3 | 3 | 0 |  | **Fazekas** | .0013 |
| 14 | 4 | 2 | 2 | 0 |  | **14** | 6 | 3 | 3 | 0 |  | **Fazekas_DWM_** | .0014 |
| 15 | 2 | 1 | 1 | 0 |  |  |  |  |  |  |  | **Fazekas_PV_** | .0050 |
| 16 | 1 | 1 | 0 | 1 |  |  |  |  |  |  |  |  |  |

^(a)^ Sub-components of the Fazekas total scores (Fazekas, range 0-6) for single RSP and nRSP iNPH patients, including the deep white matter lesion load (**Fazekas_DWM_**, range 0-3) and the periventricular white matter lesion load (**Fazekas_PV_**, range 0-3). The **Fazekas_DWM_ and Fazekas_PV_ sub-scores were highly correlated** in HCs (Spearman’s rank correlation coefficient 𝜌 = 0.63 (*p* < 10^-5^)) and iNPH patients (𝜌 = 0.90 (*p* < 10^-10^)), both in the RSP and nRSP sub-groups (RSP: 𝜌 = 0.81 (*p* = .00013); nRSP: 𝜌 = 0.96 (*p* < 10^-7^)).

^(b)^ Individual differences between the Fazekas_DWM_ and Fazekas_PV_ sub-scores. Half of RSP patients (8/16) had equal lesion load in periventricular and deep white matter areas; 4 RSP patients had larger lesion load in periventricular than in deep white matter areas (Fazekas sub-score difference = -1); 4 nRSP patients had small lesion load in deep white matter areas (Fazekas_DWM_ = 0) and no lesions in periventricular white matter areas (Fazekas sub-score difference = 1).

^(c)^ Individual differences between the Fazekas_DWM_ and Fazekas_PV_ sub-scores. 93% of nRSP patients (13/14) had equal lesion load in periventricular and deep white matter areas; 1 nRSP patient had larger lesion load in periventricular than in deep white matter areas.

^(d)^ P-values and 𝜂^2^ effect sizes from RSP/nRSP group-comparisons (Mann-Whitney U-test).

^(e)^ Generalized linear model including age as covariate. Results were unchanged when adding gender or education level as covariates.

**eTable4**. **Linear regression models relating changes in waking speed after CSFTT and single gait, neuropsychological and MRI variables measured at baseline**

| Characteristics | Model R^2 (a)^ | Model *p*-value^(a)^ | Beta^(a)^  [95% confidence interval] | Beta p-value^(a)^ |
| --- | --- | --- | --- | --- |
| A𝛽_1-42_ | 0.149 | .38 | 0.080 [-0.009, 0.170] | .077 |
| pTau | 0.068 | .77 | 0.044 [-0.050, 0.138] | .35 |
| tTau | 0.042 | .89 | 0.0215 [-0.072, 0.115] | .64 |
| Disease duration | 0.042 | .56 | -0.040 [-0.128, 0.048] | .36 |
| Walking speed | 0.126 | .16 | 0.080 [-0.168, 0.007] | .070 |
| Step length | 0.076 | .35 | 0.060 [-0.150, 0.030] | .18 |
| Step width | 0.054 | .47 | 0.050 [-0.143, 0.043] | .28 |
| TUG | 0.130 | .15 | 0.083 [-0.006, 0.171] | .065 |
| Categorical verbal fluency | 0.052 | .49 | 0.046 [-0.134, 0.042] | .29 |
| WAIS-III symbol digit modalities ^(b)^ | 0.076 | .39 | 0.034 [-0.058, 0.127] | .45 |
| FCSRT immediate free recall ^(c)^ | 0.061 | .52 | 0.028 [-0.048, 0.104] | .45 |
| Starkstein apathy scores ^(d)^ | 0.128 | .18 | 0.061 [-0.025, 0.147] | .16 |
| Relative ventricle volume | 0.043 | .89 | 0.023 [-0.071, 0.116] | .62 |
| Relative PCMF volume | 0.063 | .79 | 0.045 [-0.061, 0.151] | .39 |
| Relative CF volume | 0.118 | .51 | 0.070 [-0.164, 0.023] | .13 |
| ODI PLIC^(e)^ | 0.163 | .37 | -0.089 [-0.177, -0.002] | .046 |
| Vic PLIC^(e)^ | 0.269 | .11 | -0.140 [-0.240, -0.040] | .008 |
| ODI CING^(e)^ | 0.024 | .96 | 0.031 [-0.120, 0.057] | .47 |
| Vic CING^(e)^ | 0.162 | .38 | -0.102 [-0.202, -0.001] | .047 |
| DMN_intra_ | 0.048 | .86 | 0.028 [-0.121, 0.065] | .54 |
| DMN_SV_ | 0.046 | .87 | 0.027 [-0.068, 0.122] | .56 |
| DMN_EC_ | 0.033 | .92 | 0.002 [-0.093, 0.097] | .97 |
| Perf Thalamus^(f)^ | 0.015 | .98 | 0.008 [-0.107, 0.091] | .87 |
| Perf PCC^(f)^ | 0.154 | .40 | 0.089 [-0.006, 0.185] | .065 |
| Fazekas | 0.200 | .21 | -0.094 [-0.179, -0.009] | .031 |

^(a)^ Multi-variable linear regression analysis with relative change of *walking speed* after CSFTT as dependent variable, and a clinical or neuroimaging variable as independent variable. Age was added as covariate to the model. Independent variables were standardized before fitting the model. The coefficient of determination of the whole model (R^2^ in %), the *p*-value of the whole-model, the beta coefficient for the clinical or neuroimaging variable of interest, and the *p*-value of the related beta coefficient are reported. None of the correlations survives multiple comparison correction.

^(b)^ WAIS-III symbol digit modalities score was missing for 6 iNPH patients (2 RSP, 4 nRSP).

^(c)^ FCSRT immediate free recall score was missing for 3 iNPH patients (2 RSP, 1 nRSP) and 1 HC.

^(d)^ Starkstein apathy score was missing for 2 iNPH patients (1 RSP, 1 nRSP).

^(e)^ DWI data were missing for 2 iNPH patients (nRSP).

^(f)^ ASL data were missing for 2 iNPH patients (nRSP) and 1 HC.

**eTable5**. **Linear regression models relating changes in TUG after CSFTT and single gait, neuropsychological and MRI variables measured at baseline**

| Characteristics | Model R^2 (a)^ | Model *p*-value^(a)^ | Beta^(a)^  [95% confidence interval] | Beta p-value^(a)^ |
| --- | --- | --- | --- | --- |
| A𝛽_1-42_ | 0.267 | .029 | 0.029 [-0.032, 0.091] | .33 |
| pTau | 0.241 | .13 | 0.010 [-0.053, 0.073] | .75 |
| tTau | 0.244 | .12 | 0.013 [-0.048, 0.075] | .66 |
| Disease duration | 0.160 | .095 | -0.036 [-0.097, 0.025] | .24 |
| Walking speed | 0.195 | .053 | 0.050 [-0.012, 0.112] | .11 |
| Step length | 0.223 | .033 | 0.057 [-0.003, 0.118] | .063 |
| Step width | 0.196 | .052 | 0.051 [-0.012, 0.114] | .11 |
| TUG | 0.282 | .011 | -0.073 [-0.132, -0.013] | .018 |
| Categorical verbal fluency | 0.123 | .17 | -0.014 [-0.077, 0.049] | .65 |
| WAIS-III symbol digit modalities ^(b)^ | 0.164 | .15 | 0.003 [-0.060, 0.067] | .91 |
| FCSRT immediate free recall ^(c)^ | 0.191 | .078 | -0.019 [-0.082, 0.043] | .53 |
| Starkstein apathy scores ^(d)^ | 0.186 | .076 | -0.025 [-0.089, 0.039] | .42 |
| Relative ventricle volume | 0.253 | .11 | -0.021 [-0.082, 0.041] | .50 |
| Relative PCMF volume | 0.244 | .12 | -0.015 [-0.086, 0.055] | .66 |
| Relative CF volume | 0.275 | .080 | 0.034 [-0.028, 0.097] | .27 |
| ODI PLIC^(e)^ | 0.249 | .14 | -0.001 [-0.068, 0.065] | .97 |
| Vic PLIC^(e)^ | 0.264 | .12 | 0.027 [-0.053, 0.107] | .50 |
| ODI CING^(e)^ | 0.269 | .11 | -0.023 [-0.084, 0.037] | .43 |
| Vic CING^(e)^ | 0.305 | .069 | 0.048 [-0.025, 0.121] | .19 |
| DMN_intra_ | 0.242 | .13 | 0.010 [-0.051, 0.072] | .73 |
| DMN_SV_ | 0.240 | .13 | -0.007 [-0.070, 0.055] | .82 |
| DMN_EC_ | 0.239 | .13 | -0.003 [-0.065, 0.059] | .92 |
| Perf Thalamus^(f)^ | 0.227 | .17 | -0.001 [-0.065, 0.064] | .99 |
| Perf PCC^(f)^ | 0.254 | .14 | -0.030 [-0.097, 0.036] | .35 |
| Fazekas | 0.238 | .13 | -0.002 [-0.064, 0.059] | .94 |

^(a)^ Multi-variable linear regression analysis with relative change of *TUG* after CSFTT as dependent variable, and a clinical or neuroimaging variable as independent variable. Age was added as covariate to the model. Independent variables were standardized before fitting the model. The coefficient of determination of the whole model (R^2^ in %), the *p*-value of the whole-model, the beta coefficient for the clinical or neuroimaging variable of interest, and the *p*-value of the related beta coefficient are reported. None of the correlations survives multiple comparison correction.

^(b)^ WAIS-III symbol digit modalities score was missing for 6 iNPH patients (2 RSP, 4 nRSP).

^(c)^ FCSRT immediate free recall score was missing for 3 iNPH patients (2 RSP, 1 nRSP) and 1 HC.

^(d)^ Starkstein apathy score was missing for 2 iNPH patients (1 RSP, 1 nRSP).

^(e)^ DWI data were missing for 2 iNPH patients (nRSP).

^(f)^ ASL data were missing for 2 iNPH patients (nRSP) and 1 HC.

**eFigure1**. In the iNPH sample included in this study, 16 patients (53%) responded positively to the CSFTT. Out of these 16 RSP, 2 improved in walking speed only; 4 improved in TUG only; 10 improved in both walking speed and TUG. 14 patients did not respond to CSFTT. The scatter plot represents the percentage improvement in walking speed (*x*-axis) and TUG (*y*-axis) with respect to baseline values, for CSFTT responders (blue dots) and CSFTT non-responders (orange dot). The vertical and horizontal dashed lines represent the 10% improvement threshold on the walking speed and TUG, respectively.

**
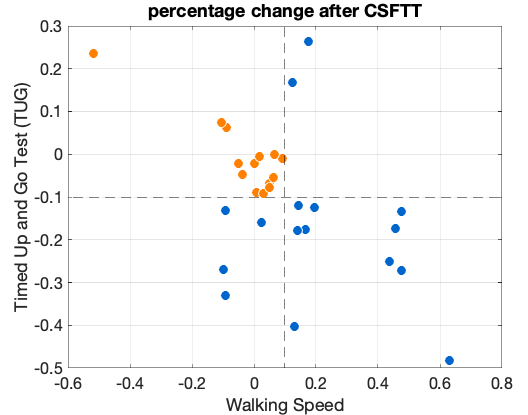
**

**eFigure2**. Spearman’s rank correlation coefficients between the 22 clinical and neuroimaging parameters, and age and education level, in all iNPH patients, RSP only and nRSP only. Asterisks indicate correlations with *p*-value < .005. None of the correlations survives Bonferroni correction.


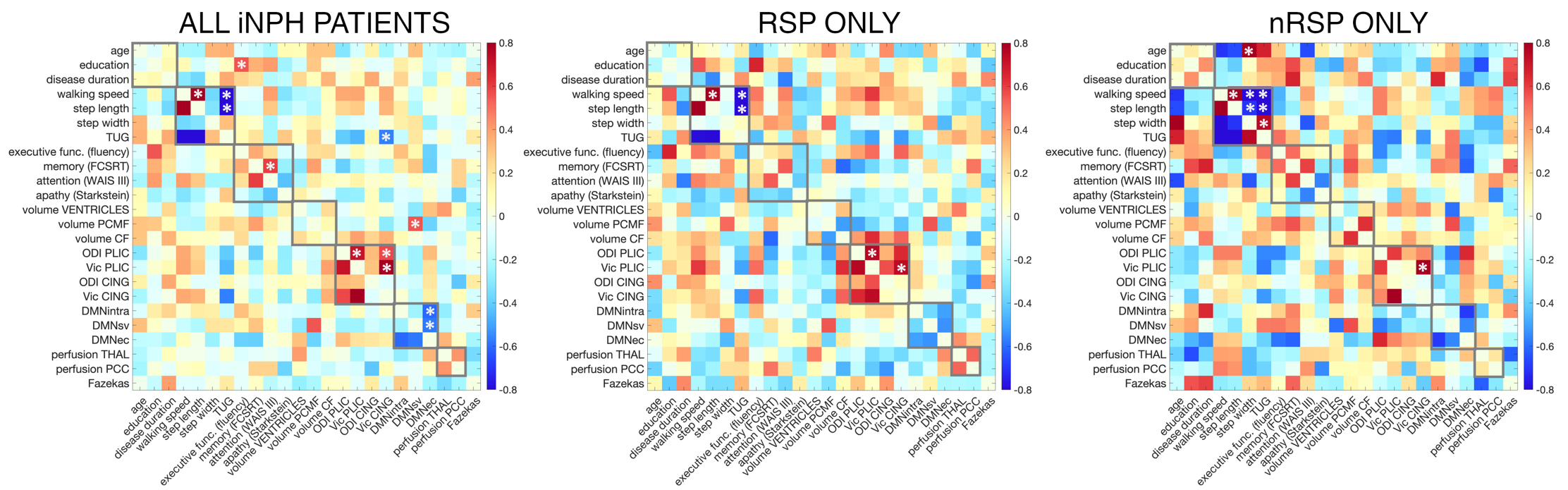

Supplement: Supplementary file 1 — Supplementary file1 (DOCX 608 KB) [file 415_2022_11168_MOESM1_ESM.docx]
